# Spring viremia of carp virus infection induces hypoxia response in zebrafish by stabilizing hif1α

**DOI:** 10.1128/jvi.01491-24

**Published:** 2024-11-27

**Authors:** Zixuan Wang, Chunchun Zhu, Xueyi Sun, Hongyan Deng, Wen Liu, Shuke Jia, Yao Bai, Wuhan Xiao, Xing Liu

**Affiliations:** 1 Key Laboratory of Breeding Biotechnology and Sustainable Aquaculture, Institute of Hydrobiology, Chinese Academy of Sciences 53021 , Wuhan, China; 2 University of the Chinese Academy of Sciences 74519 , Beijing, China; 3 College of Life Science, Wuhan University 12390 , Wuhan, China; 4 Hubei Hongshan Laboratory, Wuhan, China; University of Kentucky College of Medicine, Lexington, Kentucky, USA

**Keywords:** SVCV, virus infection, hif1α, hypoxia response, zebrafish

## Abstract

The hypoxia signaling pathway controls hypoxia adaptation and tolerance of organisms, which is regulated by multiple mechanisms. Viral infection elicits various pathophysiological responses in the host. However, whether viral infection can affect the hypoxia response is not yet fully understood. In this study, we found that Spring viremia of carp virus (SVCV) infection in zebrafish caused symptoms similar to those in zebrafish under hypoxic conditions. Further assays indicated that SVCV infection activated the hypoxia signaling pathway in zebrafish. In addition, SVCV infection caused increased glycolysis and reactive oxygen species (ROS) levels in cells. Mechanistically, SVCV-G protein interacted with hif1α-a/b and attenuated their K48-linked polyubiquitination, leading to their stabilization and subsequent enhancement of target gene expression. Moreover, treatment with the HIF1α-specific inhibitor PX478 enhanced the antiviral ability against SVCV infection in zebrafish and zebrafish cells. This study reveals a relationship between SVCV infection and the hypoxia signaling pathway in fish and provides a strategy for reducing the damage of viral disease in the aquaculture industry.

**IMPORTANCE:**

Viral infection triggers various pathophysiological responses in the host. The hypoxia signaling pathway controls hypoxia adaptation and tolerance of organisms. However, whether viral infection can affect the hypoxia response is not yet fully understood. This study showed that Spring viremia of carp virus (SVCV) infection activated the hypoxia signaling pathway and induced a hypoxia response. The SVCV-G protein interacted with hif1α-a/b and reduced their K48-linked polyubiquitination, leading to their stabilization and subsequent enhancement of target gene expression. Additionally, treatment with the HIF1α-specific inhibitor PX478 enhanced the antiviral ability against SVCV infection in zebrafish and zebrafish cells. Our findings not only reveal a relationship between SVCV infection and the hypoxia signaling pathway in fish but also provide a strategy for reducing the damage of viral disease in the aquaculture industry.

## INTRODUCTION

Oxygen is essential for the life of aerobic organisms on Earth (1). Surviving hypoxia is one of the most critical stresses faced by organisms (2, 3). Fish, as a specific group of aquatic vertebrates, have been exposed to more frequent changes in oxygen levels in their environment. Due to biotic or abiotic effects, a sudden decrease in dissolved oxygen in the water causes them to suffer severe pathophysiological damage or even death (4). Demonstrating the phenomena and the underlying mechanisms of hypoxia adaptation and tolerance in fish will not only advance our knowledge of the regulation of hypoxia signaling but also benefit the aquaculture industry.

Similar to that in mammals, the hypoxia signaling pathway is the main regulator of hypoxia adaptation and tolerance in fish (4–6). In this pathway, hypoxia-inducible factors (HIF1/2α) serve as master regulators (7). Under normoxic conditions, the HIFα subunit is hydroxylated at two highly conserved prolyl residues by the prolyl hydroxylases (PHD1, PHD2, and PHD3) (also called EglN2, EglN1, and EglN3), whose activity is regulated by the availability of O_2_ (3, 8, 9). Hydroxylated HIFα generates a binding site that is recognized by the von Hippel-Lindau (pVHL) tumor suppressor protein complex (10). This complex polyubiquitylates HIFα and targets it for proteasomal degradation. Under hypoxic conditions, the activity of PHDs is inhibited, resulting in the stabilization and accumulation of HIFα proteins. Stabilized HIFα proteins dimerize with HIF1β proteins, translocate to the nucleus, and induce transcription of genes involved in various biological processes (3, 11). Oxygen-dependent hydroxylation is an important regulatory pathway for HIFα activation and is responsible for the cellular oxygen sensing mechanism (3, 9). In addition to hydroxylation, HIFα is also regulated by other post-translational modifications (PTMs), including ubiquitination/deubiquitination, phosphorylation, acetylation/deacetylation, SUMOylation, methylation, S-nitrosylation, glycosylation, and neddylation (12–17). Most of these modifications cause either an increase or decrease in the stability of HIFα, leading to various biological consequences (18–31).

In nature, fish are also threatened by various pathogens in their environment, such as viruses. Viral infection causes various pathophysiological changes in fish, including oxygen uptake. Whether viral infection can directly affect the hypoxia signaling pathway to influence fish hypoxia response is still elusive. Spring viremia of carp virus (SVCV), a single-stranded RNA virus belonging to the family *Rhabdoviridae* (32), has been defined as the causative agent of spring viremia of carp (SVC), which causes tremendous losses in carp aquaculture worldwide (33–35). The SVCV genome encodes five viral proteins consisting of a nuclear protein (SVCV-N), a phosphoprotein (SVCV-P), a glycoprotein (SVCV-G), a matrix protein (SVCV-M), and a polymerase protein (SVCV-L) (36–39). It has been shown that SVCV has evolved different strategies to evade the host antiviral immunity by directly interfering with the function of host molecules involved in antiviral innate immunity. SVCV-N protein targets the mitochondrial antiviral-signaling protein (MAVS) to attenuate interferon (IFN) production (40). In addition, SVCV-N protein can suppress the activity of the kinase TANK-binding kinase 1 (TBK1), leading to the inhibition of subsequent IRF3 phosphorylation and activation (41). SVCV-N protein also promotes the degradation of the stimulator of IFN genes (STING) in an autophagy-lysosome-dependent manner, resulting in the attenuation of IFN production (42). SVCV-M protein suppresses the K63-linked ubiquitination of TRAF3, thereby inhibiting the interaction between MAVS and TRAF3 (43). Interestingly, SVCV has been shown to regulate zebrafish p53 function through two distinct mechanisms: SVCV-N protein promotes zebrafish p53 degradation by suppressing K63-linked ubiquitination of p53, whereas SVCV-P protein stabilizes p53 by enhancing K63-linked ubiquitination of p53 (38).

When we performed SVCV infection studies in zebrafish, we noticed an interesting phenomenon. Compared with uninfected zebrafish, infected zebrafish swam at the water surface with flared gill covers and increased breathing rate, which is very similar to the phenotype of fish under hypoxic conditions. To elucidate the mechanism underlying this phenomenon, we further investigated the relationship between SVCV infection and modulation of the hypoxia signaling pathway. In this study, we found that SVCV infection enhanced hypoxia signaling, resulting in increased glycolysis and reactive oxygen species (ROS) levels. SVCV-G protein interacts with zebrafish hif1α-a/b and attenuates their K48-linked polyubiquitination, leading to their stabilization and subsequent enhanced transcriptional activation. Furthermore, treatment with the HIF1α-specific inhibitor PX478 enhances the antiviral ability against SVCV infection in zebrafish and zebrafish cells. These results shed light on the interaction between SVCV infection and host hypoxia response and provide a potential treatment against SVCV infection in fish.

## RESULTS

### SVCV infection causes hypoxia-induced symptoms and promotes hypoxia-inducible gene expression in zebrafish

**Fig 1:**
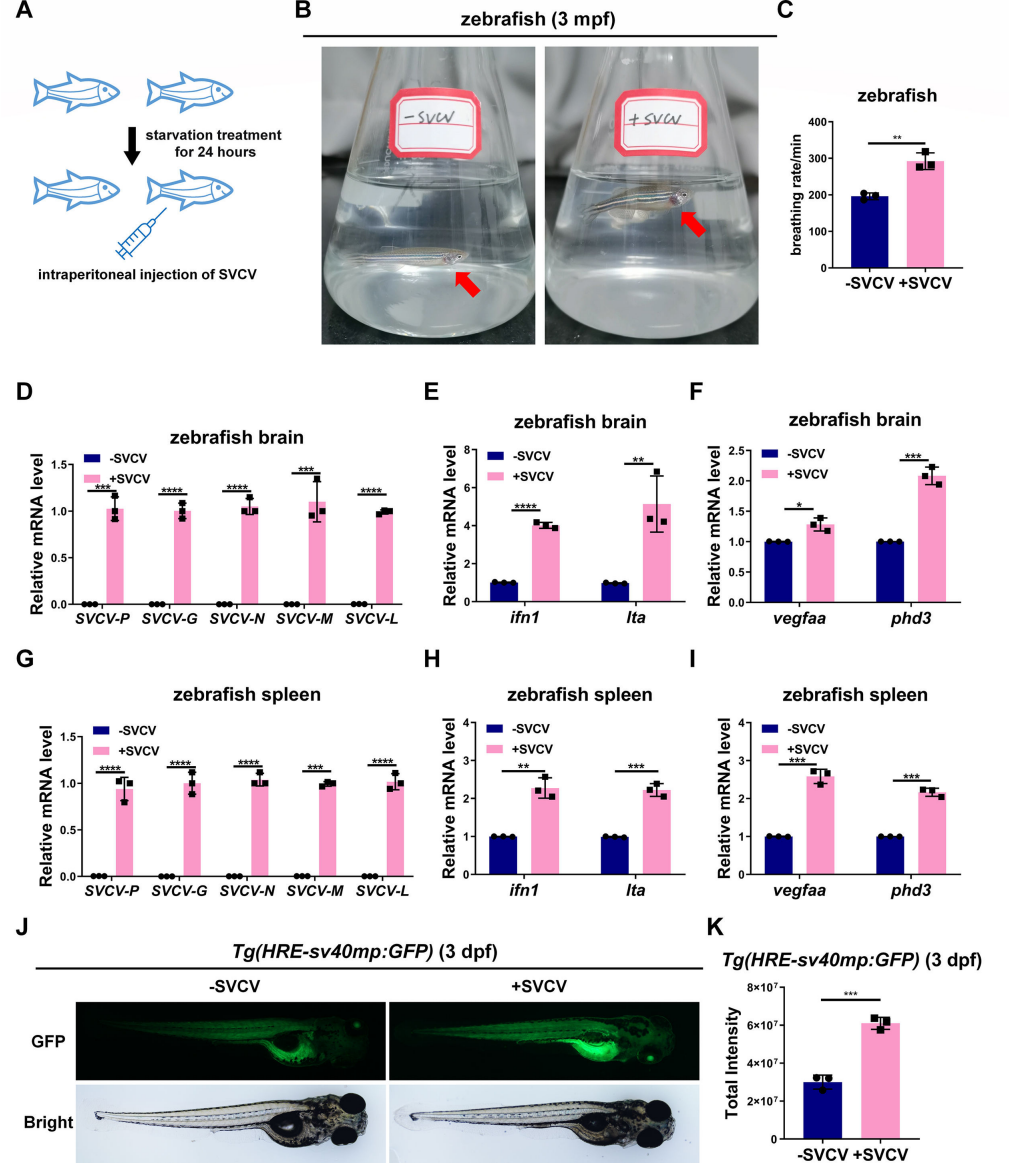
SVCV infection causes hypoxia-induced symptoms and promotes hypoxia-inducible gene expression in zebrafish. (**A-C**) SVCV infection caused symptoms in zebrafish, similar to those observed under hypoxic conditions. After 24 h of starvation, adult zebrafish (3 mpf) were injected intraperitoneally with SVCV (5 × 10^7^ TCID_50_/mL, 10 µL/individual). Twenty-four hours later, infected zebrafish were observed swimming at the surface of the water with their opercula erect and breathing rapidly. Based on the video recordings, we counted the number of times the gill covers opened and closed in three groups of zebrafish. (**D**) qPCR analysis of *SVCV-P*, *SVCV-G*, *SVCV-N*, *SVCV-M*, and *SVCV-L* mRNA in the brain of zebrafish (3 mpf) with or without infection with SVCV (5 × 10^7^ TCID_50_/mL, 10 µL/individual). (**E**) qPCR analysis of *ifn1* and *lta* mRNA in the brain of zebrafish (3 mpf) with or without infection with SVCV (5 × 10^7^ TCID_50_/mL, 10 µL/individual). (**F**) qPCR analysis of *vegfaa* and *phd3* mRNA in the brain of zebrafish (3 mpf) with or without infection with SVCV (5 × 10^7^ TCID_50_/mL, 10 µL/individual). (**G**) qPCR analysis of *SVCV-P*, *SVCV-G*, *SVCV-N*, *SVCV-M*, and *SVCV-L* mRNA in the spleen of zebrafish (3 mpf) with or without infection with SVCV (5 × 10^7^ TCID_50_/mL, 10 µL/individual). (**H**) qPCR analysis of *ifn1* and *lta* mRNA in the spleen of zebrafish (3 mpf) with or without infection with SVCV (5 × 10^7^ TCID_50_/mL, 10 µL/individual). (**I**) qPCR analysis of *vegfaa* and *phd3* mRNA in the spleen of zebrafish (3 mpf) with or without infection with SVCV (5 × 10^7^ TCID_50_/mL, 10 µL/individual). (**J**) Representative images of Tg (*HRE-sv40mp* : GFP) zebrafish larvae (3 dpf) infected with or without infection with SVCV (5 × 10^7^ TCID_50_/mL). (**K**) Quantitation of total intensity in (**J**).

To observe the behaviors of zebrafish without or with SVCV infection, we intraperitoneally injected PBS as control or SVCV into adult zebrafish (3 months post-fertilization, 3 mpf) and put them into parallel flasks (Fig. 1A). Zebrafish injected with PBS freely swam in the flask as usual (Fig. 1B, left; Video S1). However, zebrafish injected with SVCV swam at the water surface with increased breathing rate (Fig. 1B, right and C; Video S1). After a while, the infected zebrafish’s gill covers were flared (Fig. 1B, right; Video S1). The experiment was repeated several times with consistent results (Fig. S1A). Moreover, we conducted H&E staining of tissue sections to confirm the hypoxia phenotype in zebrafish upon viral infection. This revealed smaller ventricular outflow tracts in the heart and a reduction in luminal space within the central ventricular cavity. Additionally, the presence of vacuoles was observed in the interstitial hepatic tissues, whereas the gill lamellae exhibited hyperplasia, hypertrophy, and terminal dilation (Fig. S1B). These symptoms were quite similar to zebrafish that lived under hypoxic conditions or with a reduction of hypoxia tolerance (5, 6, 44, 45). By examining expression of SVCV protein genes, and antiviral responsive genes in brain and spleen, we confirmed that zebrafish were indeed successfully infected by SVCV after intraperitoneally injected with SVCV (Fig. 1D, E, G, and H). To determine whether the hypoxia signaling was affected by SVCV infection, we examined two typical hypoxia-responsive genes in zebrafish, *vegfaa* and *phd3* (5), and found that *vegfaa* and *phd3* were upregulated in zebrafish with SVCV infection compared with uninfected zebrafish (Fig. 1F and I). Furthermore, we employed the transgenic zebrafish line, Tg (*HRE-sv40mp*:GFP) in which the fluorescent protein, GFP, is driven by the promoter containing hypoxia-responsive element (HRE) (46), and observed that zebrafish larvae (*HRE-sv40mp* : GFP) (3 days post-fertilization, [dpf]) displayed a significantly higher fluorescent signal induced by SVCV infection (Fig. 1J and K). These data suggest that SVCV infection causes hypoxia-induced phenotypes in zebrafish.

### SVCV infection enhances the hypoxia signaling pathway in zebrafish and zebrafish cells

**Fig 2:**
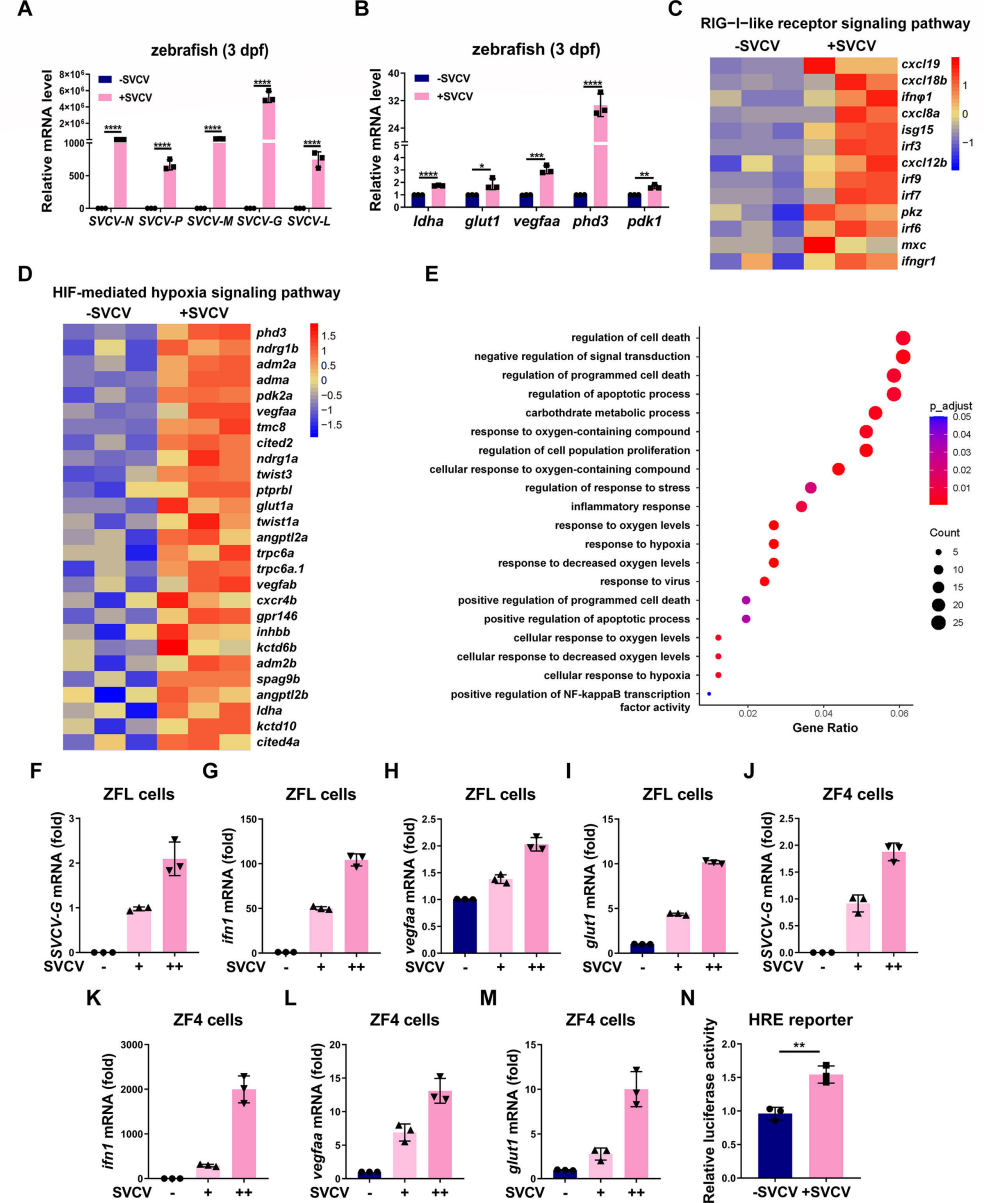
SVCV infection enhances hypoxia signaling in zebrafish and zebrafish cells. (**A**) qPCR analysis of *SVCV-N*, *SVCV-P*, *SVCV-M*, *SVCV-G*, and *SVCV-L* mRNA in zebrafish larvae (3 dpf) with or without infection with SVCV (5 × 10^7^ TCID_50_/mL). (**B**) qPCR analysis of *ldha*, *glut1*, *vegfaa*, *phd3*, and *pdk1* mRNA in zebrafish larvae (3 dpf) with or without infection with SVCV (5 × 10^7^ TCID_50_/mL). (**C**) Heatmap for the selected responsive genes of RIG-I-like receptor signaling pathway in zebrafish larvae (3 dpf) with or without infection with SVCV (5 × 10^7^ TCID_50_/mL). (**D**) Heatmap for the selected responsive genes of HIF-mediated hypoxia signaling pathway in zebrafish larvae (3 dpf) with or without infection with SVCV (5 × 10^7^ TCID_50_/mL). (**E**) Gene Ontology (GO) enrichment analyses for the DEGs were performed using the cluster Profiler version 3.8. in zebrafish larvae (3 dpf) with or without infection with SVCV (5 × 10^7^ TCID_50_/mL). (**F**) qPCR analysis of *SVCV-G* mRNA in ZFL cells infected with an increasing amount of SVCV. (**G**) qPCR analysis of *ifn1* mRNA in ZFL cells infected with an increasing amount of SVCV. (**H, I**) qPCR analysis of *vegfaa* (**H**) and *glut1* (**I**) mRNA in ZFL cells infected with an increasing amount of SVCV. (**J**) qPCR analysis of *SVCV-G* mRNA in ZF4 cells infected with an increasing amount of SVCV. (**K**) qPCR analysis of *ifn1* mRNA in ZF4 cells infected with an increasing amount of SVCV. (**L, M**) qPCR analysis of *vegfaa* (**L**) and *glut1* (**M**) mRNA in ZF4 cells infected with an increasing amount of SVCV. (**N**) HRE reporter activity in ZF4 cells with or without infection of SVCV.

Then, we investigated whether SVCV infection could indeed affect hypoxia signaling. When zebrafish larvae (3 dpf) were successfully infected by SVCV as indicated by the expression of SVCV genes (Fig. 2A), the mRNA levels of typical hypoxia-responsible genes, including *ldha*, *glut1*, *vegfaa*, *phd3*, and *pdk1*, were significantly upregulated (Fig. 2B). RNA-sequencing and transcriptomic analysis further confirmed that the genes involved in both RLR signaling pathway and hypoxia signaling pathway were enriched in SVCV-infected zebrafish larvae (Fig. 2C and D). Gene Ontology (GO) enrichment analyses revealed that several biological processes were enriched in SVCV-infected zebrafish larvae (Fig. 2E). Among these processes, the responses to viral infection and antiviral immunity were highly enriched, further validating that viral infection was achieved successfully (Fig. 2E). Interestingly, the responses to oxygen levels and hypoxia processes were also enriched in SVCV-infected zebrafish larvae (Fig. 2E). However, we found that the mRNA levels of *hif1αa* and *hif1αb* did not change significantly after SVCV infection (Fig. S2A through C). These data suggest that SVCV infection enhances the hypoxia signaling pathway in zebrafish.

Subsequently, we used zebrafish cell lines to further investigate the effect of SVCV infection on hypoxia signaling. The successful infection of SVCV on ZFL and ZF4 cell lines was confirmed by qRT-PCR analysis of the mRNA levels of G, N, P, M, or L protein of SVCV (Fig. 2F and J; Fig. S3A through H) as well as antiviral genes, *ifn1* and *mxc* (Fig. 2G and K; Fig. S3I). As expected, the mRNA levels of typical hypoxia-responsible genes, including *vegfaa* and *glut1*, were significantly upregulated in SVCV-infected ZFL cells (Fig. 2H and I). Similarly, in ZF4 cells, the mRNA levels of *vegfaa*, *glut1*, *ldha*, and *phd3*, four typical hypoxia-responsive genes, were also significantly upregulated after SVCV infection (Fig. 2L and M; Fig. S3J and K). Moreover, the activity of the HRE reporter was enhanced in SVCV-infected ZF4 cells compared with that in uninfected ZF4 cells (Fig. 2N).

### SVCV infection enhances glycolysis and increases ROS levels in zebrafish cell lines

**Fig 3:**
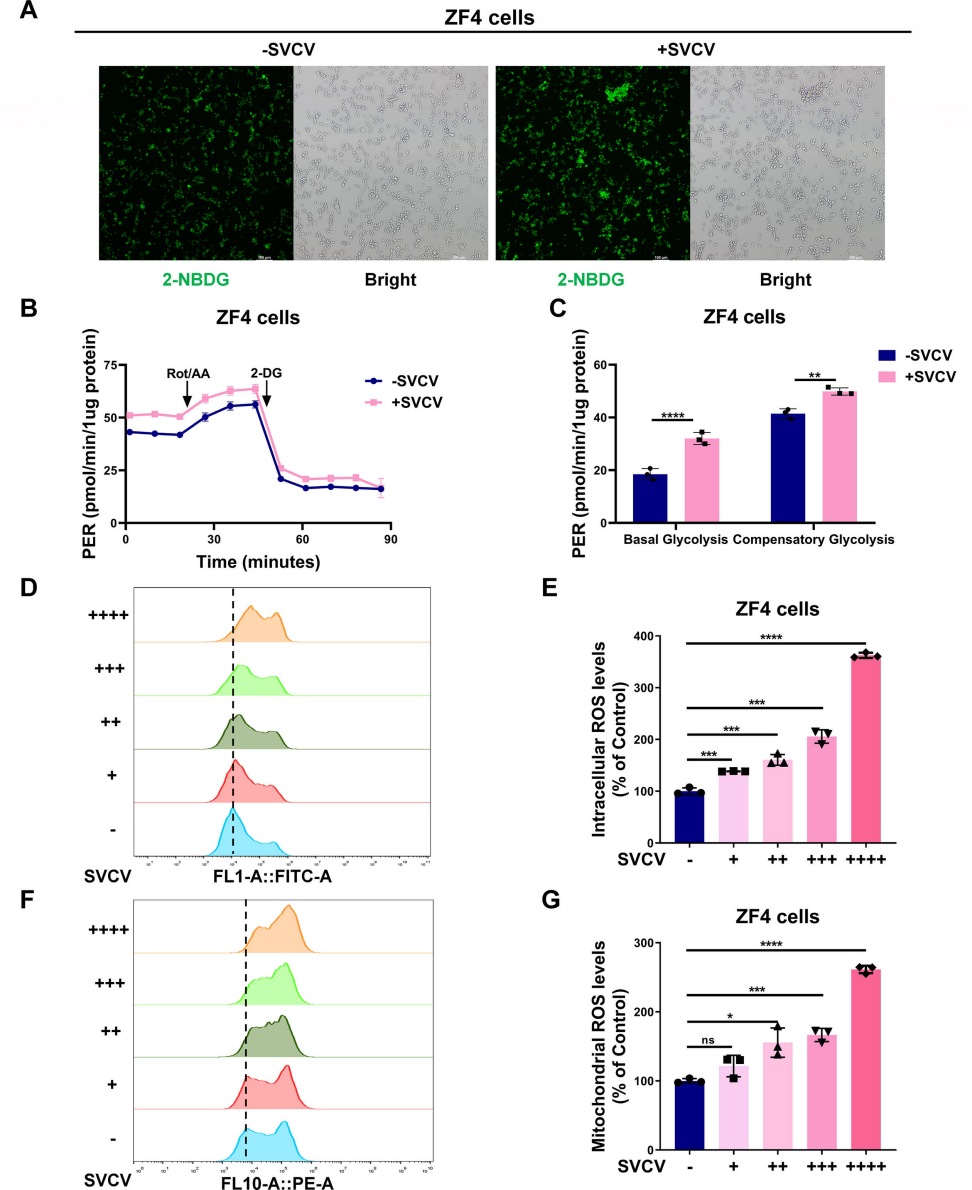
SVCV infection enhances glucose uptake, glycolytic rate, and intracellular and mitochondrial ROS levels in ZF4 cells. (**A**) Glucose uptake in ZF4 cells with or without SVCV infection as detected by uptake of the fluorescent glucose analog 2-NBDG using fluorescence microscopy (*n* = 3). Scale bar = 100 µm. (**B, C**) Proton efflux rate (PER) changes in ZF4 cells (*n* = 3) with or without SVCV infection as measured using Seahorse XFe24 Extracellular Flux Analyzer (**B**). Statistical analysis of basal glycolysis and compensatory glycolysis were shown in (**C**). (**D, E**) The levels of intracellular ROS in ZF4 cells infected with SVCV with increasing doses were detected by flow cytometry analysis. Quantitation of intracellular ROS levels in (**D**) was shown in (**E**). (**F, G**) The levels of mitochondrial ROS in ZF4 cells infected with SVCV with increasing doses were detected by flow cytometry analysis. Quantitation of mitochondrial ROS levels in (**F**) was shown in (**G**).

The well-defined biological consequence of HIF-mediated hypoxia signaling pathway is to modulate metabolic processes (47, 48). To determine the biological consequence of SVCV infection on hypoxia signaling, we initially examined the effect of SVCV infection on glucose uptake. As shown in Fig. 3A, SVCV infection greatly increased glucose uptake as revealed by fluorescence microscopy. This finding was validated by flow cytometry analysis (Fig. S4A and B). We next determined the effect of SVCV infection on glycolysis *via* the proton efflux rate measurement. SVCV infection resulted in increased basal glycolysis and compensatory glycolysis significantly (Fig. 3B and C).

The reduction of cytotoxic ROS levels correlates with cellular hypoxia adaptation and dysregulation of mitochondrial ROS levels contributes to hypoxia-induced cell apoptosis (49–52). We then investigated the effect of SVCV infection on ROS accumulation. After ZF4 cells were infected with increasing doses of SVCV, intracellular ROS levels and mitochondrial ROS levels were increased steadily (Fig. 3D through G).

Collectively, these data suggest that SVCV infection enhances the hypoxia signaling pathway, leading to increased glycolysis and elevated ROS levels in zebrafish cells.

### SVCV-G protein stabilizes hif1α

**Fig 4:**
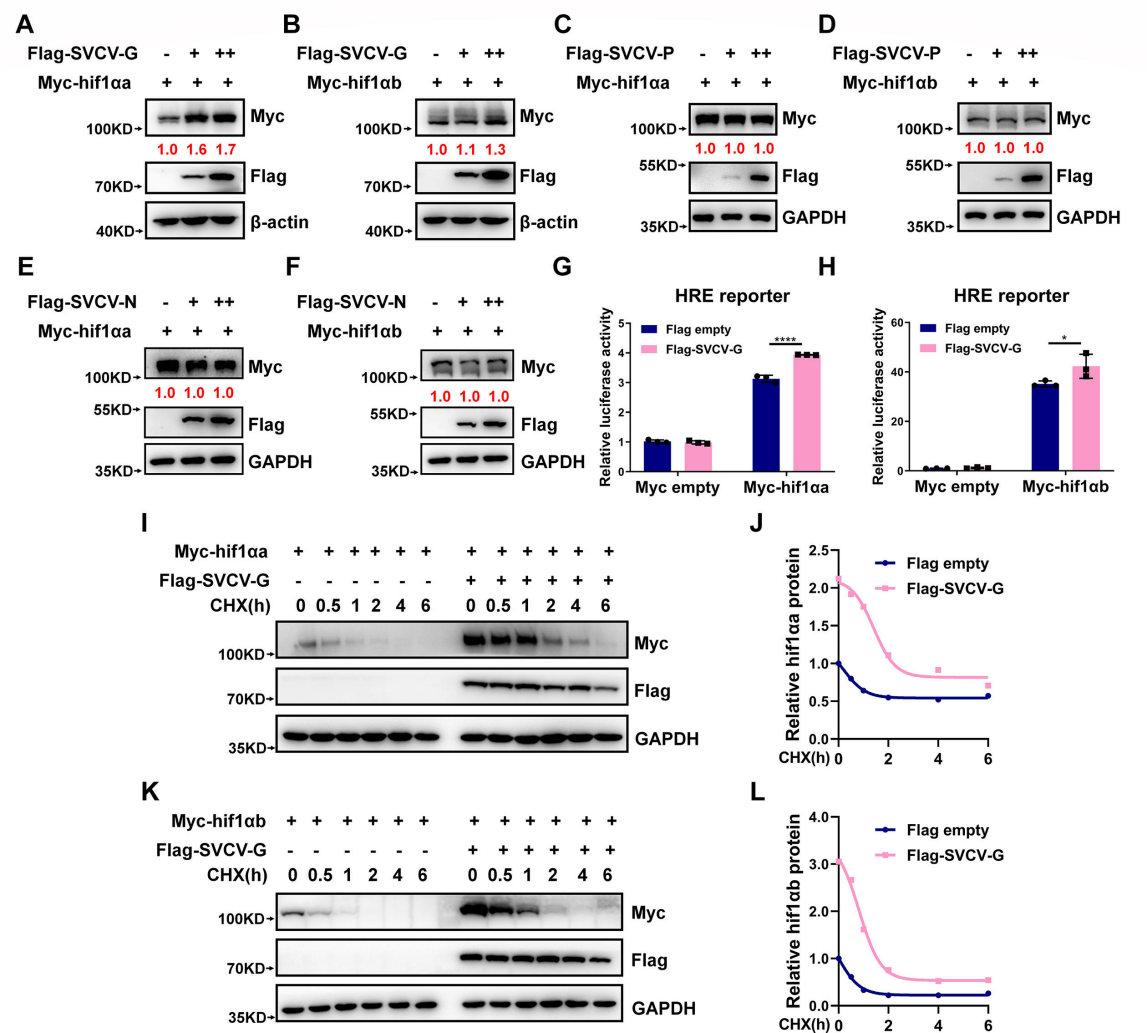
SVCV-G protein stabilizes hif1αa and hif1αb. (**A, B**) Immunoblotting (IB) of Myc-hif1αa (**A**) and Myc-hif1αb (**B**) in HEK293T cells transfected with the plasmid expressing Myc-hif1αa or Myc-hif1αb together with increasing amounts of Flag-SVCV-G (Flag empty vector [-] was used as a control). (**C, D**) IB of Myc-hif1αa (**C**) and Myc-hif1αb (**D**) in HEK293T cells transfected with the plasmid expressing Myc-hif1αa or Myc-hif1αb together with increasing amounts of Flag-SVCV-P (Flag empty vector [-] was used as a control). (**E, F**) IB of Myc-hif1αa (**E**) and Myc-hif1αb (**F**) in HEK293T cells transfected with the plasmid expressing Myc-hif1αa or Myc-hif1αb together with increasing amounts of Flag-SVCV-N (Flag empty vector [-] was used as a control). (**G, H**) HRE reporter activity in HEK293T cells transfected with the plasmid expressing Myc-hif1αa (**G**) or Myc-hif1αb (**H**) together with Flag-SVCV-G (Flag empty vector was used as a control). (**I, J**) IB of the indicated proteins in HEK293T cells transfected with Myc-hif1αa together with Flag-SVCV-G for 24 h (Flag empty vector [-] was used as a control) and then treated with cycloheximide (CHX, 50 µg/mL) for an increasing time (0–6 h) (**I**). The relative intensities of Myc-hif1αa were determined by normalizing the intensities of Myc-hif1αa to the intensities of GAPDH (**J**). (**K, L**) IB of the indicated proteins in 293T cells transfected with Myc-hif1αb together with Flag-SVCV-G for 24 h (Flag empty vector [-] was used as a control) and then treated with CHX (50 µg/mL) for an increasing time (0–6 h) (**K**). The relative intensities of Myc-hif1αb were determined by normalizing the intensities of Myc-hif1αb to the intensities of GAPDH (**L**).

To determine the mechanism of SVCV infection on the enhancement of the hypoxia signaling, we examined whether SVCV infection could affect HIF1α protein stability because HIF1α is one of the master transcription factors in the pathway and is relatively conserved between human, mouse, and zebrafish (Fig. S5). First, we examined whether the critical viral proteins of SVCV, including SVCV-G, SVCV-P, and SVCV-N, can affect the stability of two zebrafish HIF1α homologous genes, hif1αa and hif1αb. Overexpression of SVCV-G steadily stabilized hif1αa and hif1αb in a dose-dependent manner (Fig. 4A and B). However, overexpression of either SVCV-P or SVCV-N did not affect hif1αa and hif1αb (Fig. 4C through F). Consistently, overexpression of SVCV-G significantly enhanced hif1αa- or hif1αb-induced HRE reporter activity (Fig. 4G and H). Furthermore, to investigate the impact of SVCV on endogenous hif1α protein levels in zebrafish, we initially screened for suitable antibodies that could detect zebrafish hif1α protein (Fig. S6A) and identified one commercially available antibody that was capable of detecting zebrafish hif1α (Fig. S6A). Subsequently, we employed siRNA to knockdown SVCV-G and evaluated the protein levels of endogenous hif1α in the presence and absence of SVCV infection (Fig. S6B). As illustrated in Fig. S6B, the first and second lanes demonstrate that SVCV stabilizes endogenous HIF-1α proteins. Conversely, the stabilization is abolished after interfering with the synthesis of SVCV-G, as evidenced in the third and fourth lanes of the same figure.

We then further validated the stabilization of hif1αa and hif1αb by SVCV-G protein. By cycloheximide (CHX) treatment for blocking new protein synthesis, overexpression of SVCV-G still caused stabilization of hif-1αa and hif-1αb (Fig. 4I through L), suggesting that SVCV-G protein may directly prevent protein degradation of hif1αa or hif1αb.

These data suggest that the G protein of SVCV directly stabilizes zebrafish hif1αa and hif1αb, leading to the enhancement of the hypoxia signaling.

### SVCV-G protein attenuates K48-linked polyubiquitination of zebrafish hif1α

**Fig 5:**
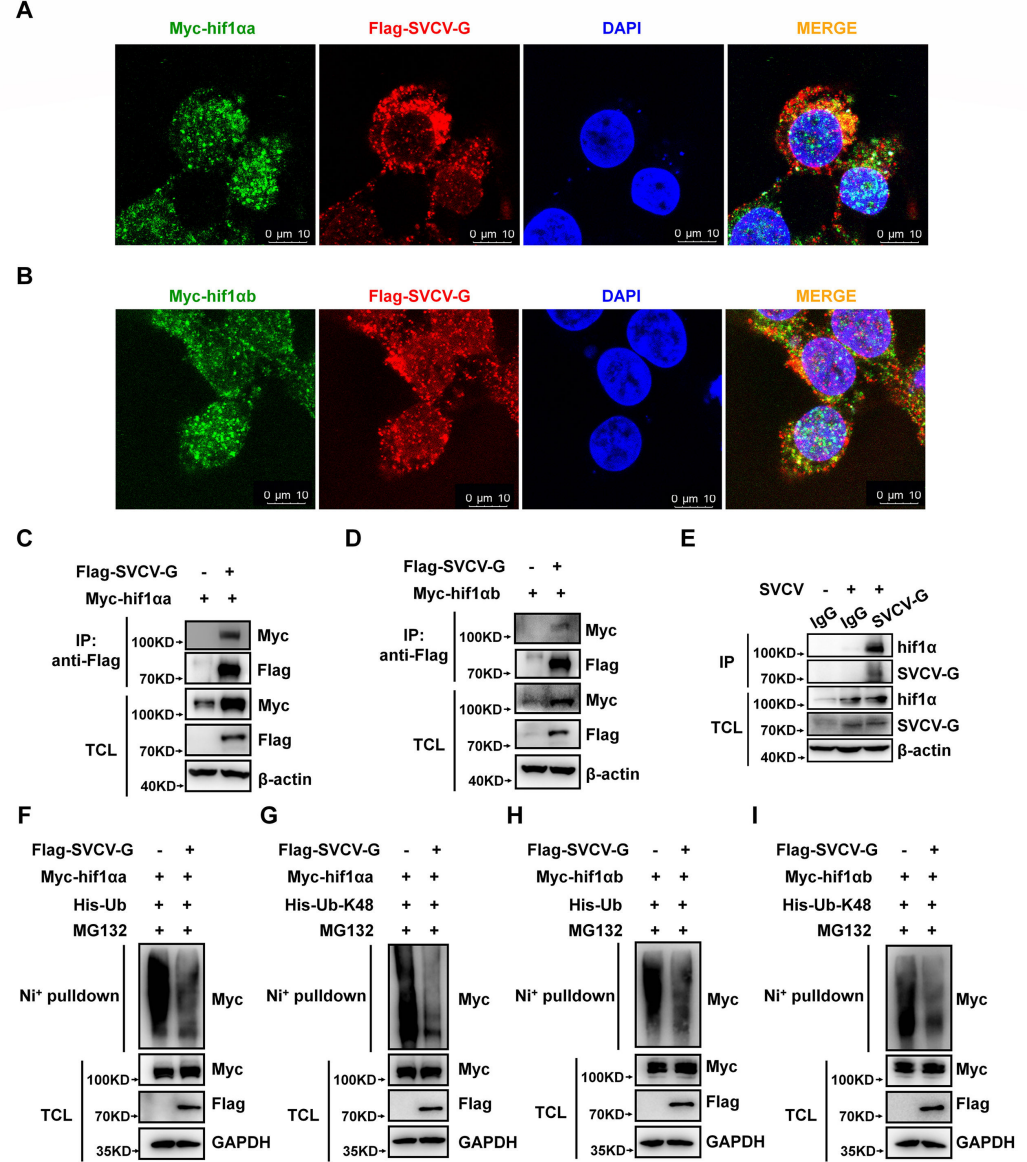
SVCV-G protein interacts with hif1αa and hif1αb to attenuate K48-linked polyubiquitination of hif1αa and hif1αb. (**A**) Myc-hif1αa co-localized with Flag-SVCV-G as revealed by co-localization assays. Scale bar = 10 µm. (**B**) Myc-hif1αb co-localized with Flag-SVCV-G as revealed by co-localization assays. Scale bar = 10 µm. (**C**) Myc-hif1αa interacted with Flag-SVCV-G as revealed by co-immunoprecipitation assays. (**D**) Myc-hif1αb interacted with Flag-SVCV-G as revealed by co-immunoprecipitation assays. (**E**) Endogenous interaction between hif1α and SVCV in ZFL cells. Anti-SVCV-G antibody was used for immunoprecipitation (IP), and the interaction was detected by IB using anti-hif1α antibody. (**F, G**) Ubiquitination analysis of hif1αa in HEK293T cells transfected with Myc-hif1αa, Flag-SVCV-G (Flag empty vector [-] was used as a control), and His-Ub (**F**) or His-Ub-K48 (**G**) for 24 h, and then treated with MG132 (20 µM) for 8 h. (**H, I**) Ubiquitination analysis of hif1αb in HEK293T cells transfected with Myc-hif1αb, Flag-SVCV-G (Flag empty vector [-] was used as a control), and His-Ub (**H**) or His-Ub-K48(I) for 24 h, and then treated with MG132 (20 µM) for 8 h.

To understand the mechanisms of SVCV-G on the stabilization of zebrafish hif1α, we performed a series of assays. Co-localization assay indicated that ectopically expressed SVCV-G could co-localize with ectopically expressed hif1αa or hif1αb protein (Fig. 5A and B). Then, we transfected Myc-hif1αa or Myc-hif1αb together with or without Flag-SVCV-G into HEK293T cells and found that ectopic expression of SVCV-G interacted with ectopically expressed hif1αa or hif1αb protein (Fig. 5C and D). After ZFL cells were infected with or without SVCV for 24 h, anti-SVCV-G antibody was used for immunoprecipitation, and endogenous hif1α was found to interact with SVCV-G in ZFL cells (Fig. 5E).

We then examined whether SVCV-G could affect the ubiquitination of hif1αa or hif1αb. We blocked the degradation of hif1αa or hif1αb using MG-132 and found that overexpression of SVCV-G in HEK293T cells suppressed the ubiquitination of co-transfected hif1αa or hif1αb (Fig. 5F and H). Since K48-linked polyubiquitination is usually associated with protein degradation, we therefore examined the effects of SVCV-G on K48-linked polyubiquitination of hif1αa or hif1αb and found that overexpression of SVCV-G suppressed K48-linked polyubiquitination of co-transfected hif1αa or hif1αb in HEK293T cells (Fig. 5G and I).

Taken together, these data suggest that SVCV-G protein interacts with hif1αa and hif1αb to reduce K48-linked polyubiquitination of hif1αa and hif1αb, thereby stabilizing hif1αa and hif1αb and resulting in the enhancement of the hypoxia signaling.

### HIF1α inhibitor PX478 enhances the antiviral ability against SVCV infection in zebrafish and zebrafish cells

**Fig 6:**
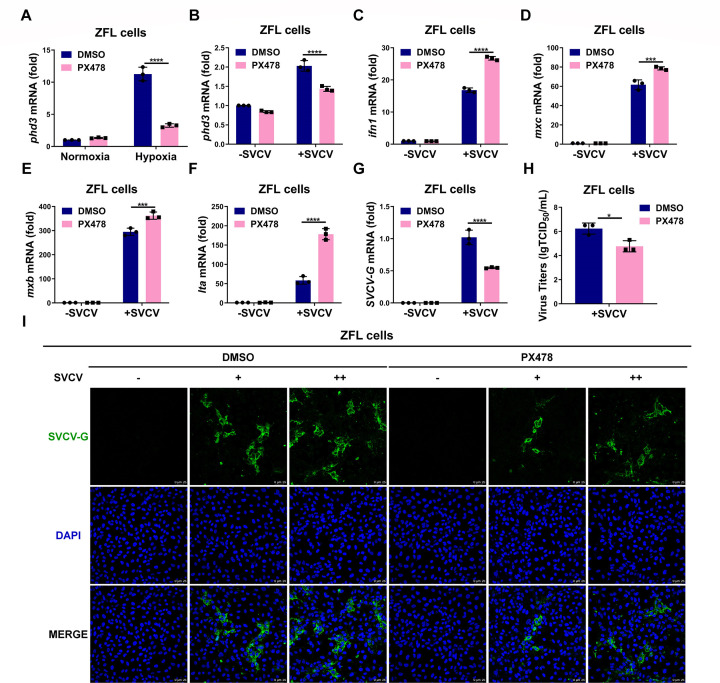
HIF-1α inhibitor PX478 enhances the antiviral ability against SVCV infection and inhibits SVCV replication in ZFL cells. (**A**) qPCR analysis of *phd3* mRNA in ZFL cells treated with PX478 (10 µM) for 12 h, followed by hypoxia treatment for 24 h. (**B**) qPCR analysis of *phd3* mRNA in ZFL cells treated with PX478 (10 µM) for 12 h, followed by infected with or without SVCV for 24 h. (**C-F**) qPCR analysis of *ifn1* (**C**), *mxc* (**D**), *mxb* (**E**), and *lta* (**F**) mRNA in ZFL cells treated with PX478 (10 µM) for 12 h, followed by infected with or without SVCV for 24 h. (**G**) qPCR analysis of *SVCV-G* mRNA in ZFL cells treated with PX478 (10 µM) for 12 h, followed by infected with or without SVCV for 24 h. (**H**) ZFL cells were treated with or without PX478 (10 µM) for 12 h, followed by those infected with SVCV for 24 h. Culture supernatant was collected, and viral titers were measured by a 50% tissue culture–infective dose (TCID_50_) assay on EPC cells. (**I**) ZFL cells seeded on 35 mm glass bottom cell culture dishes were treated with PX478 (10 µM) for 12 h, followed by infected with increased gradient SVCV for 24 h. The cells were fixed and stained with anti-SVCV-G antibody and subsequent confocal microscopy analysis. Scale bar = 25 µm.

Since SVCV infection stabilizes hif1α proteins and induces hypoxia response in zebrafish, we sought to know whether inhibiting hif1α could modulate antiviral response. Thus, we used PX478, a specific HIF1α inhibitor (53, 54), to block HIF1α activity. Initially, we confirmed that PX478 did not have an obvious effect on cell apoptosis and viability when used at a range of 10–20 µM (Fig. S7A). Moreover, PX478 was found to significantly inhibit the expression of *phd3*, a typical hypoxia-responsive gene, under hypoxia, demonstrating that PX478 indeed inhibited the HIF1α-mediated hypoxia signaling pathway (Fig. 6A). Treatment with PX478 suppressed the expression of *phd3* in SVCV-infected ZFL cells (Fig. 6B) but enhanced the expression of the key antiviral gene, including *ifn1, mxc, mxb,* and *lta* (Fig. 6C through F). In addition, the expression of five viral genes of SVCV, including *SVCV-G*, *SVCV-P*, *SVCV-N*, *SVCV-M*, and *SVCV-L,* was greatly repressed in ZFL cells upon PX478 treatment (Fig. 6G; Fig. S7B through E). Meanwhile, viral titers in ZFL cells were also repressed upon PX478 treatment (Fig. 6H). Subsequently, we used immunofluorescent staining with anti-SVCV-G antibody to detect viral proteins in SVCV-infected ZFL cells with or without PX478 treatment. Much less green signal, representing SVCV-G proteins, was observed in ZFL cells with PX478 treatment (Fig. 6I). It has been reported that PX478 can inhibit HIF1α deubiquitination, leading to increased levels of polyubiquitinated HIF1α (55). Then, we confirmed that SVCV-G overexpression suppressed K48-linked polyubiquitination of co-transfected hif1αa or hif1αb in the presence of PX478 treatment (Fig. S7F and G).

**Fig 7:**
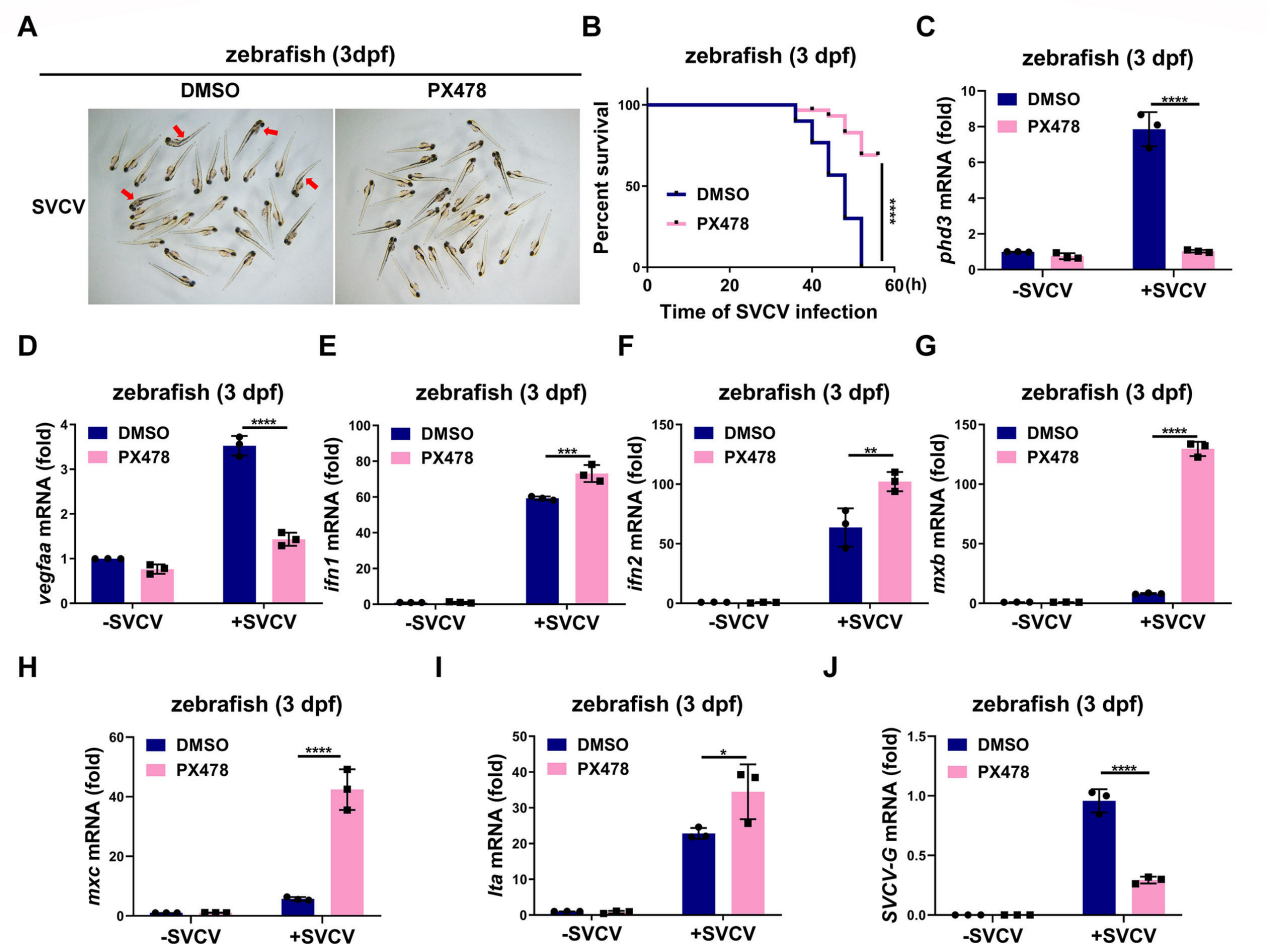
HIF-1α inhibitor PX478 enhances the antiviral ability against SVCV infection and inhibits SVCV replication in zebrafish. (**A**) Representative images of zebrafish larvae (3 dpf) treated with DMSO (vehicle control) or PX478 (10 µM) for 12 h, followed by infected with SVCV (5 × 10^7^ TCID_50_/mL) for 36 h. (**B**) Survival (Kaplan-Meier curve) of zebrafish larvae (3 dpf) (*n* = 30 per group) treated with PX478 (10 µM) for 12 h, followed by infected with or without SVCV (5 × 10^7^ TCID_50_/mL) for 52 h. (**C, D**) qPCR analysis of *phd3* (**C**) and *vegfaa* (**D**) mRNA in zebrafish larvae (3 dpf) treated with DMSO (vehicle control) or PX478 (10 µM) for 12 h, followed by infected with or without SVCV (5 × 10^7^ TCID_50_/mL) for 24 h. (**E-I**) qPCR analysis of *ifn1* (**E**), *ifn2* (**F**), *mxb* (**G**), *mxc* (**H**), and *lta* (**I**) mRNA in zebrafish larvae (3 dpf) treated with DMSO (vehicle control) or PX478(10 µM) for 12 h, followed by infected with or without SVCV (5 × 10^7^ TCID_50_/mL) for 24 h. (**J**) qPCR analysis of *SVCV-G* mRNA in zebrafish larvae (3 dpf) treated with DMSO (vehicle control) or PX478(10 µM) for 12 h, followed by infected with or without SVCV (5 × 10^7^ TCID_50_/mL) for 24 h.

Next, we sought to determine whether PX478 treatment could enhance antiviral ability against SVCV infection in zebrafish. First, we tested the toxicity of PX478 on zebrafish larvae (3 dpf) at different concentrations (Fig. S8A) and then chose 10 µM for further assays. Treatment with PX478 in zebrafish larvae led to enhanced antiviral ability against SVCV infection (Fig. 7A and B). Expression of *phd3* and *vegfaa* was decreased in PX478-treated zebrafish larvae, indicating that PX478 efficiently blocked the activity of hif1α (Fig. 7C and D). Consistently, the expression of *ifn1*, *ifn2*, *mxb*, *mxc*, and *lta* was largely induced in PX478-treated zebrafish larvae upon challenge with SVCV compared with that in vehicle control-treated zebrafish larvae (Fig. 7E through I). Moreover, the expression of five viral genes of SVCV, including *SVCV-G*, *SVCV-P*, *SVCV-N*, *SVCV-M*, and *SVCV-L,* was greatly repressed in PX478-treated zebrafish larvae compared with that in vehicle control-treated zebrafish larvae (Fig. 7J; Fig. S8B through E).

Taken together, these data suggest that suppressing hif1α with its specific inhibitor PX478 may enhance the antiviral ability against SVCV infection in zebrafish and zebrafish cells.

## DISCUSSION

The relationship between viral infection and oxygen hemostasis has been studied, but it is complicated and somewhat contradictory (56). Some viral infections activate hypoxia signaling, leading to alterations in host cellular metabolism and viral replication through various mechanisms. Hepatitis B virus X protein (HBx) stabilizes and activates HIF1α by impairing its interaction with pVHL (57). Vaccinia virus (VACV) infection causes stabilization and subsequent activation of HIF1α even under normoxic conditions, resulting from VACV protein C16 binding to PHD2 and inhibiting PHD2-mediated hydroxylation of HIF1α (58). Influenza A virus (H1N1) infection causes induction of hypoxia response by stabilizing HIF1α (59). Porcine reproductive and respiratory syndrome virus (PRRSV) infection elevates HIF1α expression and enhances viral replication (60). Recently, we found that oxygen potentiates fish anti-SVCV infection (61), and low oxygen tension (hypoxia) suppresses SVCV-induced IFN activation and enhances virus replication (61). On the other hand, the components of the hypoxia-signaling pathway have also been shown to regulate SVCV infection and antiviral immunity (61–63).VHL, the critical regulator of HIF1α ubiquitination, was found to target MAVS for ubiquitination and degradation, resulting in suppression of IFN activation (62). PHD3, an important hydroxylase for HIF1α hydroxylation and a well-defined target gene of HIF1α, was reported to repress IRF7 transactivity independent of its prolyl hydroxylase activity (63). In addition, hypoxic responses and innate immune responses have been reported to be interdependent (64–66). Lactate reduction by inactivation of lactate dehydrogenase A (LDHA), a classical hypoxia-inducible gene, increases type I IFN production to protect mice from viral infection (65). Interferon-stimulated gene 15 (ISG15), an important antiviral protein, has been reported to play a critical role in the hypoxia response (67, 68).

As aquatic vertebrates, fish face more frequent changes in environmental oxygen-dissolved water; thus, the hypoxia signaling pathway is more critical for governing fish pathophysiological reactions (35, 38, 42, 43, 61, 69). However, whether aquatic viruses can affect fish hypoxia signaling pathway remains largely unknown. In this study, we found that SVCV-G protein interacts with hif1α-a/b and inhibits their ubiquitination, leading to the stabilization of hif1αa and hif1αb proteins, providing a direct connection between viral infection and oxygen hemostasis in fish.

Based on our present study and previous studies, we have demonstrated a clear relationship between viral infection and host hypoxia signaling in fish. On one hand, low oxygen can suppress antiviral innate immunity, leading to enhanced viral replication in fish. On the other hand, viral infection leads to a reduction in hypoxia tolerance in fish. In addition, treating zebrafish with the HIF1α-specific inhibitor PX478 enhances the antiviral ability against SVCV infection, suggesting that PX478 treatment may be a potential and useful strategy to protect fish against viral infection.

## MATERIALS AND METHODS

### Zebrafish

AB line wild-type zebrafish were provided by the China Zebrafish Resource Center (CZRC). Tg (*HRE-sv40mp* : GFP) zebrafish were provided by Dr. Ling Lu (Ocean University of China). All experiments with zebrafish were approved by the Institutional Animal Care and Use Committee of the Institute of Hydrobiology, Chinese Academy of Sciences.

### Cells, viruses, and reagents

Zebrafish liver (ZFL) cells (American Type Culture Collection, ATCC) were cultivated at 28°C in 5% CO_2_ in Ham’s F12 nutrient mixture medium (Invitrogen) supplemented with 10% FBS (VivaCell). ZF4 cells (American Type Culture Collection, ATCC) were cultured at 28°C in 5% CO_2_ in DMEM: F12 (1:1) medium (VivaCell) containing 10% FBS (VivaCell). Epithelioma papulosum cyprini (EPC) cells were cultivated at 28℃ in 5% CO_2_ in medium 199 (VivaCell) plus 10% FBS (VivaCell). HEK293T cells were grown at 37°C in 5% CO_2_ in DMEM (VivaCell) supplemented with 10% FBS. The cell culture flasks, dishes, or plates were purchased from SORFA Life Science. Spring viremia of carp viruses (SVCV) were propagated in EPC cells until CPE was observed. Then, the cell culture medium with SVCV was collected and stored at −80°C until used. MG-132 (#474790) was purchased from Sigma. PX478 (#S7612) was purchased from Selleck. Cycloheximide (#HY-12320) was purchased from MCE.

### Viral infection

Three-month-old zebrafish were intraperitoneally injected with SVCV (~5.0 × 10^7^ TCID_50_/mL) at 10 µL/individual. PBS was used as the control. After 24 h of intraperitoneal injection, the brain and spleen were dissected. Zebrafish larvae were placed in disposable cell culture dishes (60 mm in diameter) containing 4 mL of egg water, and 2 mL of SVCV (~5.0 × 10^7^ TCID_50_/mL) was added to each dish. For zebrafish cells (ZFL or ZF4 cells), the cells were cultured overnight, infected with SVCV (~5.0 × 10^7^ TCID_50_/mL), and then cultivated for the indicated time, followed by the indicated assays.

### Heart, liver, and gill histology

Heart, liver, and gill tissues from zebrafish infected with or without SVCV were dissected, fixed in 10% phosphate-buffered formalin, embedded in paraffin, sectioned, stained with hematoxylin and eosin solution, and examined for histologic changes by light microscopy.

### RNA-seq

Whole RNA of zebrafish larvae (3 dpf) infected with or without SVCV was purified using RNeasy Mini Kit (QIAGEN NO. 74104). Three biological replicates were used for each sample. The transcriptome library for sequencing was generated using NEBNext Ultra RNA Library Prep Kit for Illumina (NEB, USA, #E7530L) following the manufacturer’s protocol. In order to ensure the quality of information analysis, the software *fastp* was used to remove the splice sequences, filter the low-quality, N bases (indicating that the base information could not be determined), and obtain high-quality clean data. clean data, and the number of bases and sequences of the clean data was counted. At the same time, GC, Q20, and Q30 content of the clean data was calculated (GenePlus, Beijing, China). Heatmaps were generated using Multi Experiment Viewer (MeV) software. GO enrichment analyses for the differentially expressed genes (DEGs) were performed using the cluster Profiler version 3.8.

### Quantitative real-time PCR (qPCR) assay

Total RNAs from zebrafish (brain, spleen, and larvae) or cells (ZFL and ZF4) were extracted with TransZol (#ET101-01, TransGen Biotech, China) followed by the manufacturer’s protocol. RNA quality and purity, RNA integrity, and RNA concentration were analyzed by NanoDrop Microvolume Spectrophotometers. The OD260/OD280 ratio for all RNA was in the range of 1.8–2.0. DNase I (#EN0521, Fermentas) was used to digest genomic DNA in RNAs, and then, equal amounts of RNA (1.5 µg per 20 µL reaction volume, respectively) were used for cDNA synthesis with EasyScript One-Step gDNA Removal and cDNA Synthesis SuperMix (#AE311-03, TransGen Biotech, China). MonAmp SYBR Green qPCR Mix (#RN04006M, Monad Biotech, China) was utilized for qPCR assays, and the qPCR program was set as 95°C for 5 min, 40 cycles at 95°C for 3 s, and then 60°C for 15 s, followed by melt curves. The qPCR program was run on the CFX Connect Real-Time PCR System (Bio-Rad Laboratories), with no template controls and three technical replicates. Primers used for qPCR assays are listed in Table S1.

### Glucose uptake assay

Glucose uptake was analyzed directly using the fluorescent glucose analog 2-NBDG. ZF4 cells were incubated in a glucose-free medium overnight and then were infected with or without SVCV (~5.0 × 10^7^ TCID_50_/mL) for 6 h. Fifty micrometers of 2-NBDG were added into the medium, and then, the cells were incubated for 1 h at 37°C in dark, and the amount of 2-NBDG taken up by cells was detected by fluorescence microscope and flow cytometry.

### Glycolytic rate test

The proton efflux rate (PER) under glycolytic rate test assays were performed using the Seahorse XFe24 Extracellular Flux Analyzer (Agilent Technologies, Santa Clara, CA, USA). Glycolytic rate test assays were performed using the XF Glycolytic rate Assay Kit (Agilent Technologies, #103344–100). The assays were performed according to the manufacturer’s instructions. The ZF4 cells (4 × 10^4^ cells/well) were cultured in XF24 cell culture microplate (Agilent Technologies, #102340–100) infected with or without SVCV. For glycolytic rate test assay, antimycin A and rotenone mixture (0.5 µM) and 2-deoxy-D-glucose (50 mM) were added to the cell culture plate for determining glycolytic flux including basal glycolysis and compensatory glycolysis.

### Measurement of intracellular and mitochondrial ROS levels

ZF4 cells were cultured and infected with or without SVCV as indicated. After infection, ZF4 cells were collected and counted. For intracellular ROS level measurement, cells (1 × 10^6^) were incubated in PBS solution containing 1 µM of CM-H2DCFDA (#C6827, Thermo Fisher) at 37°C for 60 min in dark and then washed with PBS three times, followed by flow cytometric analysis. For mitochondrial ROS level measurement, cells (1 × 10^6^) were incubated in PBS solution containing 5 µM of MitoSOX Red (#M36008, Thermo Fisher) at 37°C for 10 min in dark and then washed with PBS three times, followed by flow cytometric analysis.

### Western blot analysis and co-immunoprecipitation assay

Total protein of cells and immunoprecipitants was separated by 10% SDS-PAGE and transferred to polyvinylidene difluoride (PVDF) membrane (Bio-Rad). Then, the membranes were blocked for 1.5 h at room temperature in TBST buffer containing 5% non-fat milk and then were incubated overnight with primary antibody dilutions at 4°C. Then, after being washed three times with TBST buffer, the membranes were incubated with secondary antibody dilutions for 1 h at room temperature. After three additional washes with TBST buffer, the blots were photographed with the Fuji Film LAS4000 mini-luminescent image analyzer. The antibodies used in this experiment: anti–c-Myc (1:1000, #SC-40; Santa Cruz Biotechnology), anti-Flag (1:5000, #F1804; Sigma), anti–β-actin (1:20,000, #AC026; ABclonal Technology), anti-GAPDH (1:2000, #SC-477242; Santa Cruz Biotechnology), anti-HIF1α (1:1000, #36169, Cell Signaling Technology), and anti-HIF-1α (1:1000, #A6265, ABclonal). Anti-Flag antibody-conjugated agarose beads (#B23101) were purchased from Sigma. Anti-SVCV-G antibody (1:1000) was provided by Dr. Shun Li (Institute of Hydrobiology, Chinese Academy of Sciences).

### Luciferase reporter assays

HEK293T and ZF4 cells were grown in 24-well plates and transfected with various amounts of plasmids by VigoFect (Vigorous Biotech., Beijing, China), as well as with CMV-Renilla used as a control. After the cells were transfected for 24 h, the luciferase activity was determined by the Dual-Luciferase Reporter Assay System (Promega). Then, the data were normalized to *Renilla* luciferase. Data are representative of three independent experiments (mean ± SD)

### Ubiquitination assay

For analysis of the ubiquitination of hif1αa and hif1αb, HEK293T cells were transfected with plasmids expressing Flag-SVCV-G, Myc-hif1αa or Myc-hif1αb, and His-ubiquitin (His-Ub) or His-ubiquitin mutant (His-Ub-K48) for 24 h and then lysed by denatured buffer (6 M guanidine-HCl, 0.1 M Na2HPO4/NaH2PO4, 10 mM imidazole). Ubiquitination assays with His-ubiquitin were performed by affinity purification using Ni^2+^ - NTA resin (Novagen) and then immunoblotting with the indicated antibody.

### Immunofluorescent confocal microscopy

Cells seeded on 35 mm glass bottom cell culture dishes (#201200, SORFA) were cultured under the indicated treatment. Then, the treated cells were fixed with paraformaldehyde (4% in PBS) at room temperature for 30 min. After washing three times with PBS, the slides were well blocked in the buffer comprising goat serum (5%), BSA (2 mg/mL), and Triton X-100 (0.1%) in PBS at room temperature for 1 h. After that, the slides were stained with anti-Myc (1:500, #2278, Cell Signaling Technology), anti-Flag(1:1000, #F1804; Sigma), or anti-SVCV-G antibodies(1:2000) offered by Dr. Shun Li (Institute of Hydrobiology, Chinese Academy of Sciences) at room temperature for 2 h (a 1:1000 dilution for each antibody, respectively), and then, after three additional washes with PBS containing 1% BSA, it was incubated with Alexa Fluor 488 goat anti-Rabbit IgG (1:1000, #A-11008, Thermo Scientific) and/or Alexa Fluor 594 goat anti-mouse IgG (1:1000, #A-11005, Thermo Scientific) at room temperature for 1 h. Subsequently, the slides were mounted carefully using a mounting medium with DAPI and photographed using a laser scanning confocal microscope (Leica SP8, Germany).

### Statical analysis

GraphPad Prism software (8.0) was used for all statistical analysis. Results with error bars express mean ± SD. Survival data were calculated by the Kaplan-Meier method and analyzed by the log-rank test. Other statistical analyses were performed by using Student’s two-tailed *t* test (where two groups of data were compared) or two-way ANOVA analysis (where more than two groups of data were compared). A *P* value less than 0.05 was considered significant. Statistical significance is represented as follows: *P < 0.05, **P < 0.01, ***P < 0.001, ****P < 0.0001.

## Data Availability

The original RNA-seq data were uploaded to GEO under accession GSE260453. Further information and requests for resources and reagents should be directed to and will be fulfilled by Xing Liu and Wuhan Xiao.

## SUPPLEMENTAL MATERIAL

Supplemental material jvi.01491-24-s0001.pdf Supplemental legends, Figures S1 to S8, and Table S1.

Video S1 jvi.01491-24-s0002.mp4 SVCV infection causes hypoxia-induced symptoms in zebrafish.

ASM does not own the copyrights to Supplemental Material that may be linked to, or accessed through, an article. The authors have granted ASM a non-exclusive, world-wide license to publish the Supplemental Material files. Please contact the corresponding author directly for reuse.
