# Supplementary material for: Spring viremia of carp virus infection induces hypoxia response in zebrafish by stabilizing hif1α
Source: J Virol. 2024 Nov 27;99(1):e01491-24. doi: 10.1128/jvi.01491-24 (PMC11784138; doi:10.1128/jvi.01491-24)
Supplement: Supplemental material — Supplemental legends, Figures S1 to S8, and Table S1. [file jvi.01491-24-s0001.pdf]

# Supplementary information

## Supplementary Data

**Video S1. SVCV infection causes hypoxia-induced symptoms in zebrafish.** After 24 hours of starvation, adult zebrafish (3 mpf) were injected intraperitoneally with SVCV ($5 \times 10^7$ $TCID_{50}$/mL, 10 μL/individual). 24 hours later, infected zebrafish were observed swimming at the surface of the water with their opercula erect and breathing rapidly.

**Figure S1. SVCV infection causes hypoxia-induced symptoms in zebrafish.**

(A) SVCV infection caused symptoms in zebrafish, similar to those observed under hypoxic conditions. After 24 hours of starvation, adult zebrafish (3 mpf) were injected intraperitoneally with SVCV ($5 \times 10^7$ $TCID_{50}$/mL, 10 μL/individual). 24 hours later, infected zebrafish were observed swimming at the surface of the water with their opercula erect and breathing rapidly. Here are the results of six additional sets of replicated experiments.

(B) H&E staining of heart, liver and gill tissue sections of zebrafish infected with or without SVCV.

**Figure S2. SVCV infection does not affect the mRNA levels of *hif1αa* and *hif1αb* in zebrafish**

(A) Heatmap for the *hif1αa* and *hif1αb* in zebrafish larvae (3 dpf) with or without infection with SVCV ($5 \times 10^7$ $TCID_{50}$/mL).

(B) qPCR analysis of *hif1αa* mRNA in zebrafish larvae (3 dpf) with or without infection with SVCV ($5 \times 10^7$ $TCID_{50}$/mL).

(C) qPCR analysis of *hif1αb* mRNA in zebrafish larvae (3 dpf) with or without infection with SVCV ($5 \times 10^7$ $TCID_{50}$/mL).

**Figure S3. SVCV infection enhances hypoxia signaling in ZFL cells and ZF4 cells.**

(A-D) qPCR analysis of *SVCV-N* (A), *SVCV-P* (B), *SVCV-M* (C), and *SVCV-L* (D) mRNA in ZFL cells infected with an increasing amount of SVCV.

(E-H) qPCR analysis of *SVCV-N* (E), *SVCV-P* (F), *SVCV-M* (G), and *SVCV-L* (H) mRNA in ZF4 cells infected with an increasing amount of SVCV.

(I) qPCR analysis of *mxc* mRNA in ZF4 cells infected with an increasing amount of SVCV.

(J-K) qPCR analysis of *ldha* (J) and *phd3* (K) mRNA in ZF4 cells infected with an increasing amount of SVCV.

**Figure S4. SVCV infection enhances glucose uptake in ZF4 cells.**

(A) Glucose uptake in ZF4 cells infected with or without SVCV was detected using the fluorescent glucose analog, 2-NBDG, by flow cytometry analysis.

(B) Quantitation of glucose uptake in (A).

**Figure S5. Conservation of HIF1α between human, mouse and zebrafish.**

Alignment of the amino acid sequences, including human HIF1α (NP_001521.1), mouse Hif1α (NP_001300848.1), zebrafish hif1αa (NP_001295488.1), and zebrafish hif1αb (NP_001296971.1), using the SnapGene 6.0.2 program.

**Figure S6. The effects of SVCV-G protein on the protein levels of hif1α.**

(A) IB analysis in HEK293T cells transfected with the plasmid expressing Myc-hif1αa or Myc-hif1αb (Myc empty vector was used as a control), using commercial anti-HIF1α antibodies.

(B) IB of endogenous hif1α in ZFL cells transfected with SVCV-G or control siRNAs for 24 h and then infected with or without of SVCV for 24 h.

**Figure S7. The effects of HIF-1α inhibitor PX478 on SVCV replication and the effects of SVCV-G protein on the ubiquitination of hif1α with PX478 treatment.**

(A) ZFL cells were treated with PX478 (from 0 to 20 μM) for 24 h and cell apoptosis was determined by staining with PI and Annexin V. Quantitation of apoptotic cells is shown in the right panel.

(B) qPCR analysis of *SVCV-P* mRNA in ZFL cells treated with PX478 (10 μM) for 12 h, followed by infected with or without SVCV for 24 h.

(C) qPCR analysis of *SVCV-N* mRNA in ZFL cells treated with PX478 (10 μM) for 12 h, followed by infected with or without SVCV for 24 h.

(D) qPCR analysis of *SVCV-M* mRNA in ZFL cells treated with PX478 (10 μM) for 12 h, followed by infected with or without SVCV for 24 h.

(E) qPCR analysis of *SVCV-L* mRNA in ZFL cells treated with PX478 (10 μM) for 12 h, followed by infected with or without SVCV for 24 h.

(F) Ubiquitination analysis of hif1αb in HEK293T cells transfected with Myc-hif1αa, Flag-SVCV-G (Flag empty vector [-] was used as a control) and His-Ub-K48 for 24 h, and then treated with MG132 (20 μM) and PX478 (10μM) for 8 h.

(G) Ubiquitination analysis of hif1αb in HEK293T cells transfected with Myc-hif1αb, Flag-SVCV-G (Flag empty vector [-] was used as a control) and His-Ub-K48 for 24 h, and then treated with MG132 (20 μM) and PX478 (10μM) for 8 h.

**Figure S8. HIF-1α inhibitor PX478 inhibits SVCV replication in zebrafish.**

(A) Representative images of zebrafish larvae (3 dpf) treated with PX478 (from 0 to 20 μM) for 24 h.

(B) qPCR analysis of *SVCV-P* mRNA in zebrafish larvae (3 dpf) treated with DMSO (vehicle control) or PX478 (10 μM) for 12 h, followed by infected with or without SVCV ($5 \times 10^7$ $TCID_{50}$/mL) for 24 h.

(C) qPCR analysis of *SVCV-N* mRNA in zebrafish larvae (3 dpf) treated with DMSO (vehicle control) or PX478 (10 μM) for 12 h, followed by infected with or without SVCV ($5 \times 10^7$ $TCID_{50}$/mL) for 24 h.

(D) qPCR analysis of *SVCV-M* mRNA in zebrafish larvae (3 dpf) treated with DMSO (vehicle control) or PX478 (10 μM) for 12 h, followed by infected with or without SVCV ($5 \times 10^7$ $TCID_{50}$/mL) for 24 h.

(E) qPCR analysis of *SVCV-L* mRNA in zebrafish larvae (3 dpf) treated with DMSO (vehicle control) or PX478 (10 μM) for 12 h, followed by infected with or without SVCV ($5 \times 10^7$ $TCID_{50}$/mL) for 24 h.

**Figure S1**

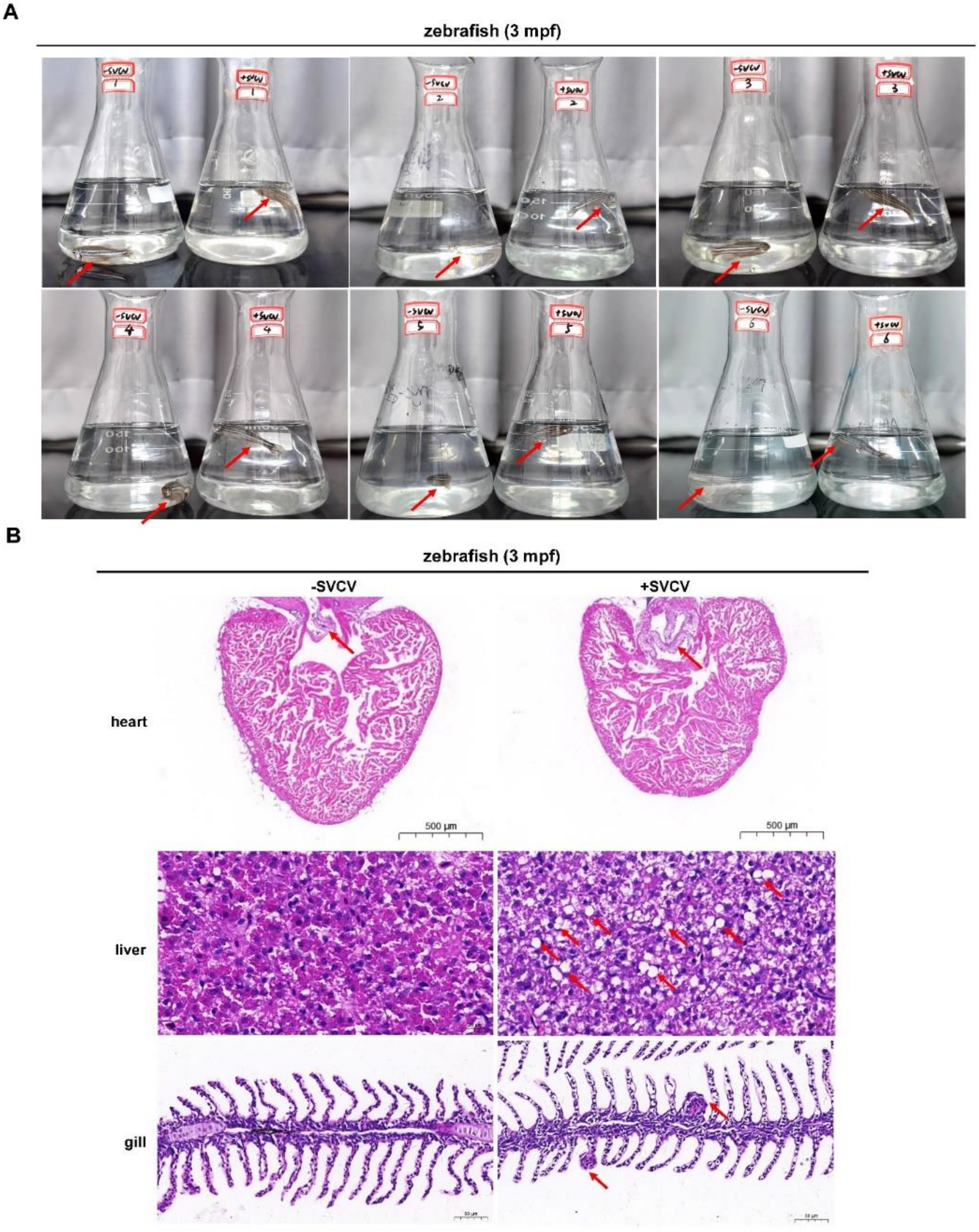
A
zebrafish (3 mpf)
B
zebrafish (3 mpf)
-SVCV
+SVCV
heart
500 µm
500 µm
liver
gill

# Figure S2

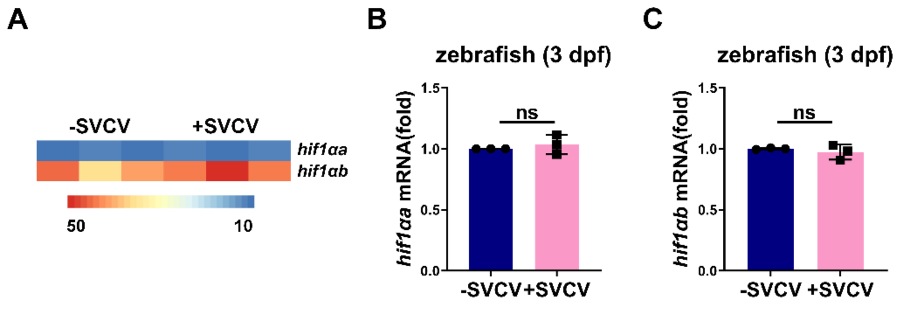


# Figure S3

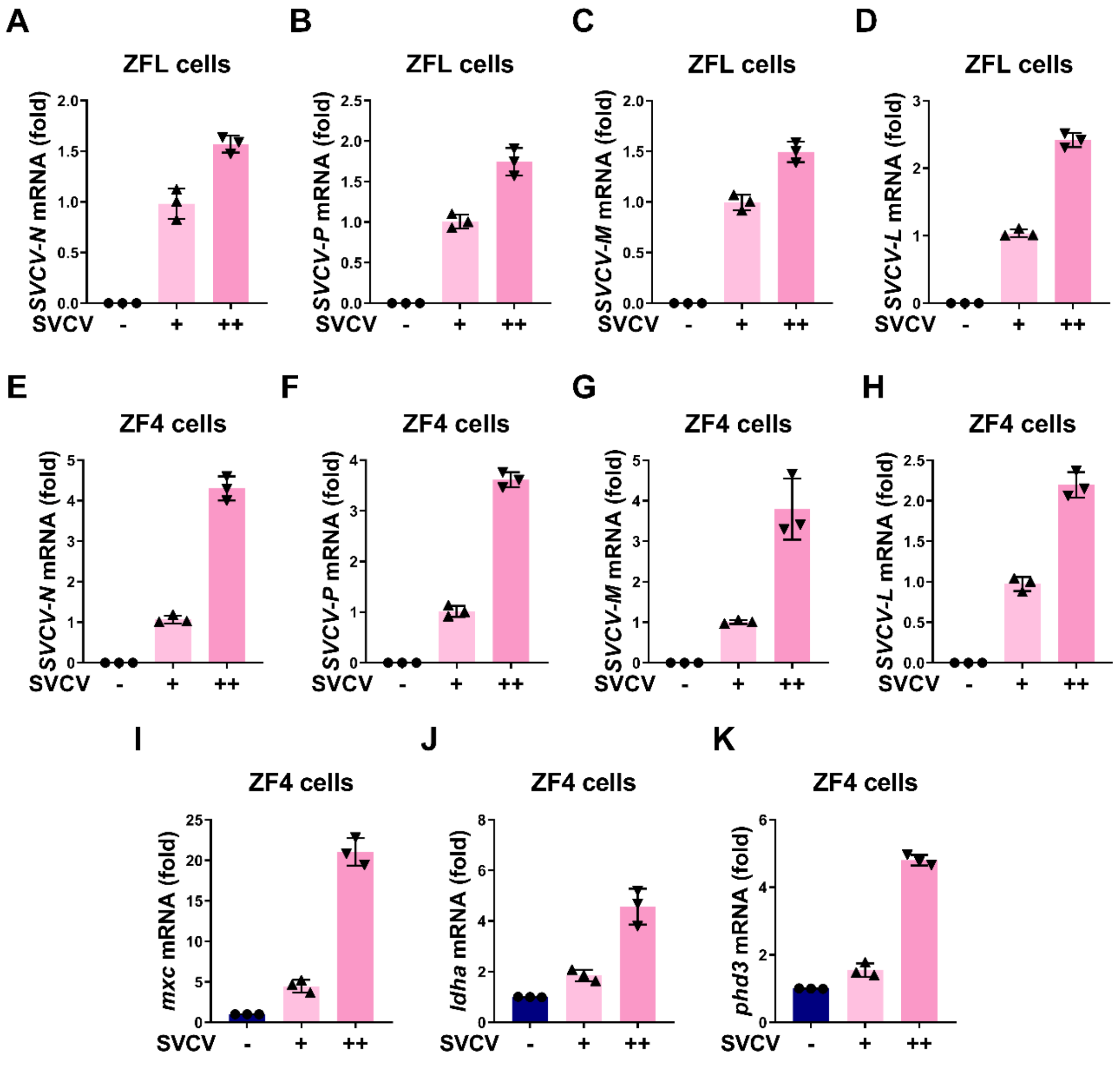

## Figure S4

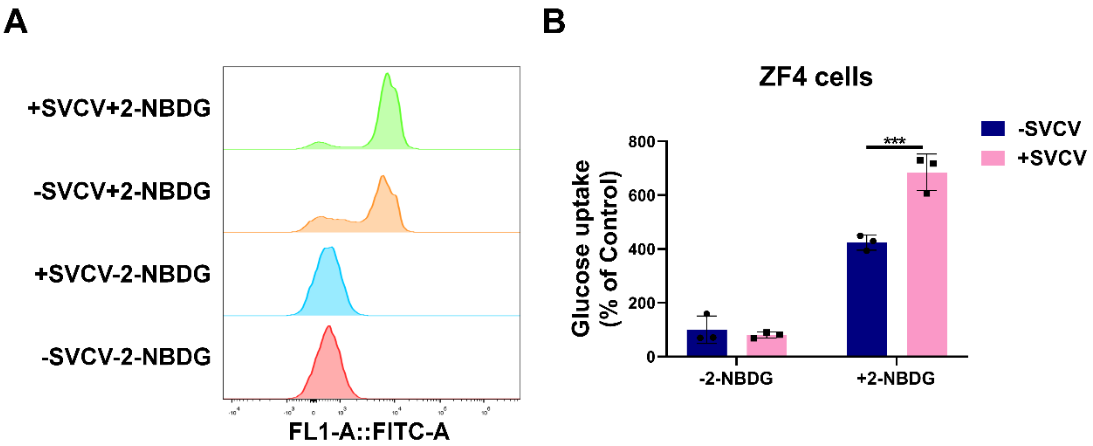


## Figure S5

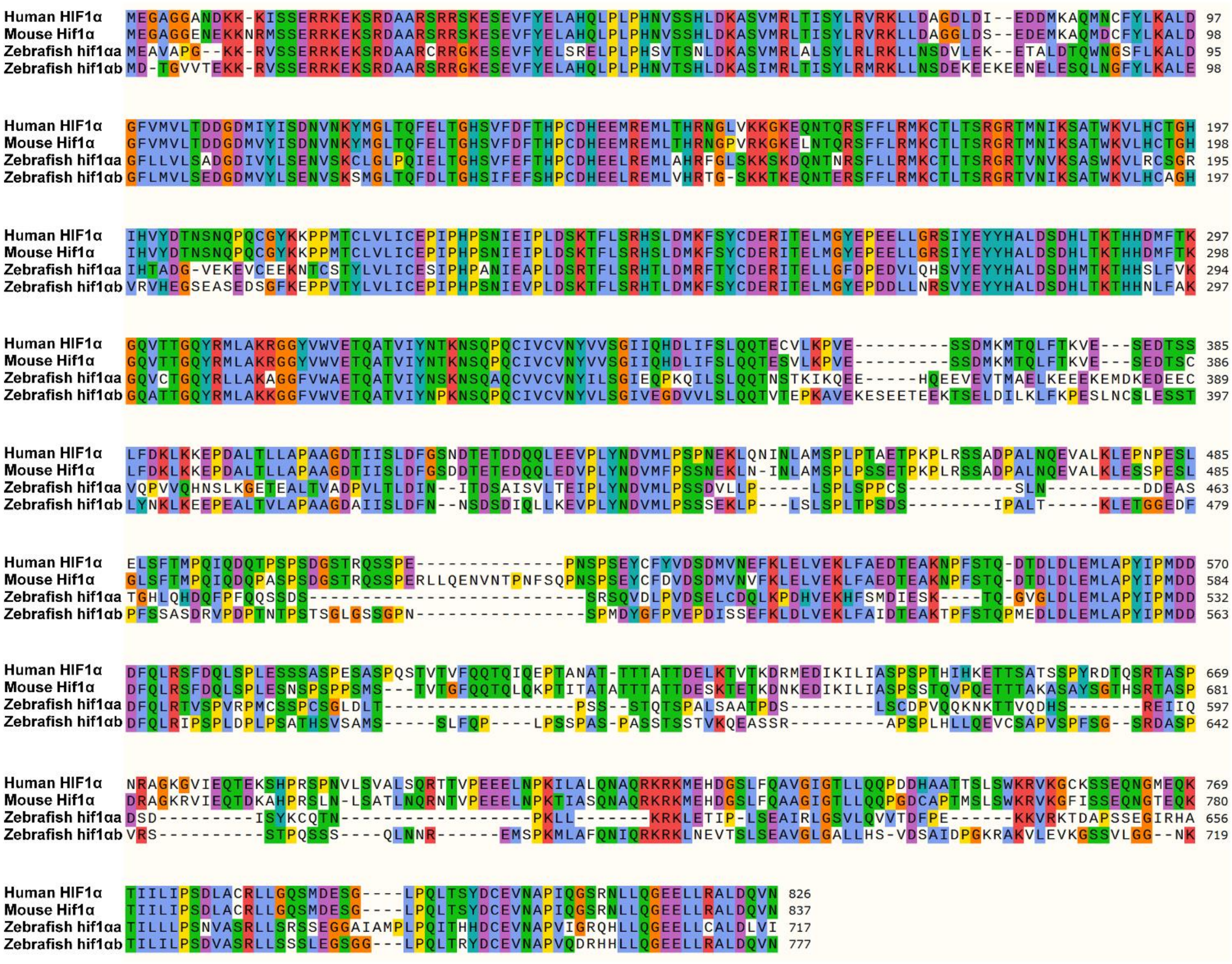

## Figure S6

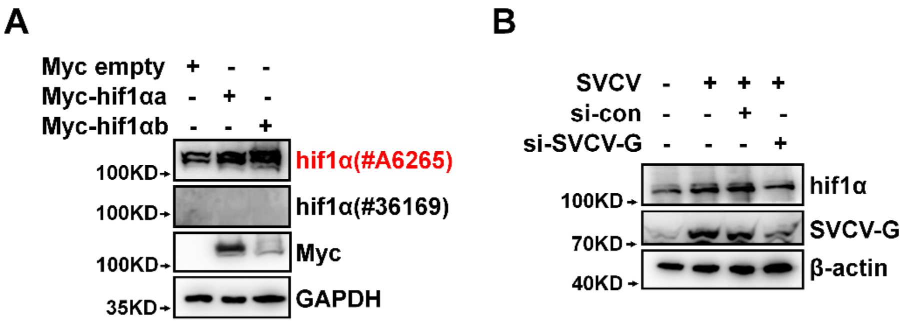


## Figure S7

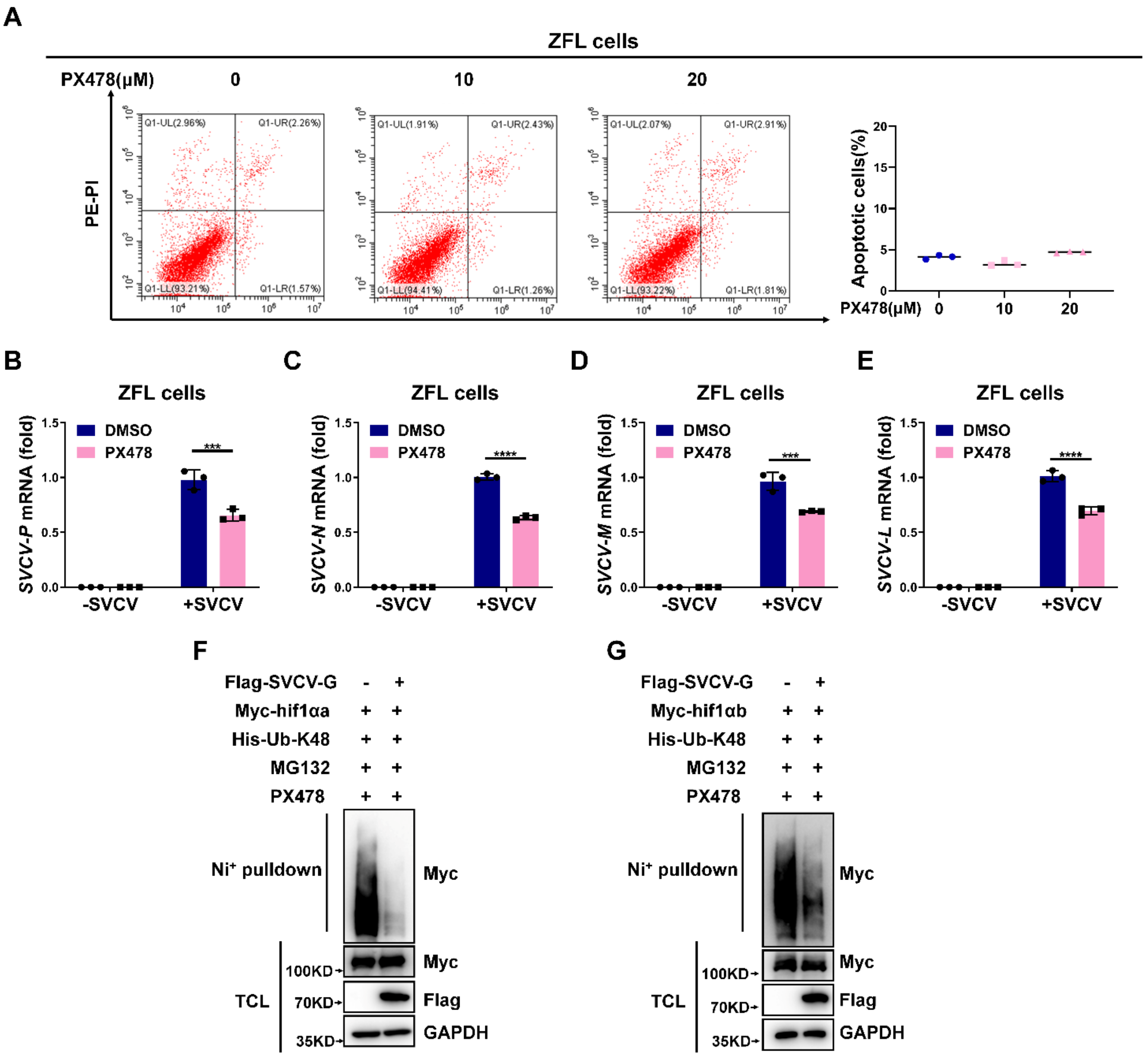

## Figure S8

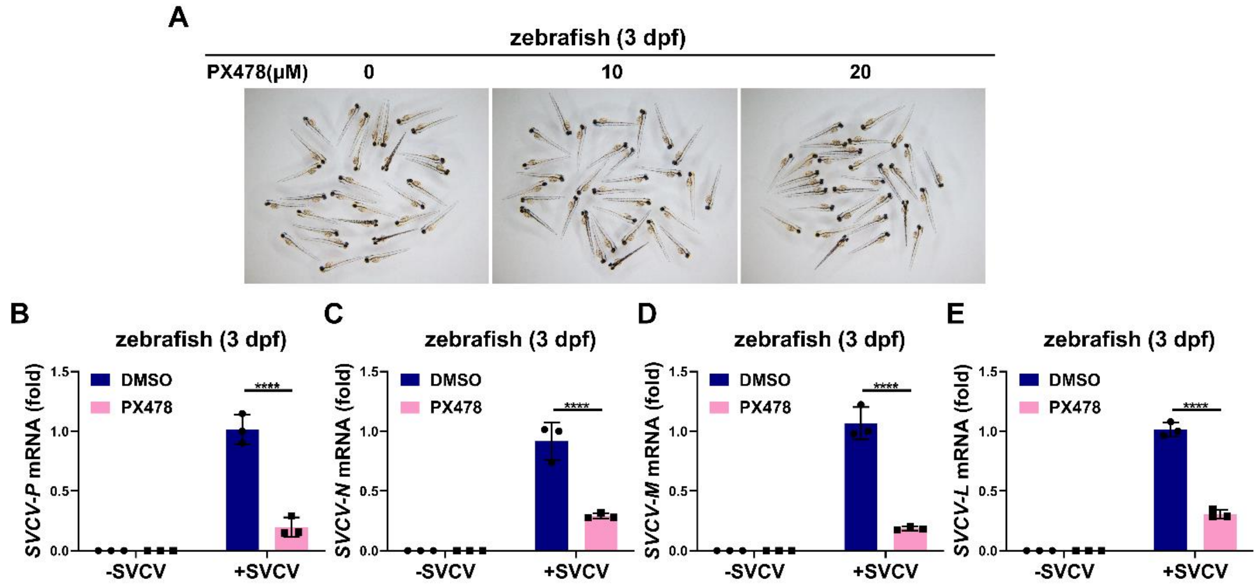

A
zebrafish (3 dpf)
PX478(μM)
0
10
20
B
C
D
E
zebrafish (3 dpf)
SVCV-P mRNA (fold)
SVCV-N mRNA (fold)
SVCV-M mRNA (fold)
SVCV-L mRNA (fold)
DMSO
PX478
****
0.0
0.5
1.0
1.5
-SVCV
+SVCV

**Supplemental Table S1. The primer sequences**

| Primers | Sequence (5' to 3') |
|---|---|
| *SVCV-N*-RT | F: TGAGGTGAGTGCTGAGGATG |
| | R: CCATCAGCAAAGTCCGGTAT |
| *SVCV-P*-RT | F: TTGGACCTGGGATAGTGA |
| | R: CTTGCTTGGTTTGTGGG |
| *SVCV-G*-RT | F: CGACCTGGATTAGACTTG |
| | R: AATGTTCCGTTTCTCACT |
| *SVCV-M*-RT | F: TACTCCTCCCACTTACGA |
| | R: CAAGAGTCCGAGAAGGTC |
| *SVCV-L*-RT | F: GCCCACTTTGCATCCAGTCC |
| | R: GCCCACTTTGCATCCAGTCC |
| zebrafish *β-actin* (internal control)-RT | F: TACAATGAGCTCCGTGTTGC |
| | R: ACATACATGGCAGGGGTGTT |
| zebrafish *ifn1*-RT | F: GAGCACATGAACTCGGTGAA |
| | R: TGCGTATCTTGCCACACATT |
| zebrafish *ifn2*-RT | F: CCTCTTTGCCAACGACAGTT |
| | R: CGGTTCCTTGAGCTCTCATC |
| zebrafish *mxb*-RT | F: AATGGTGATCCGCTATCTGC |
| | R: TCTGGCGGCTCAGTAAGTTT |
| zebrafish *lta*-RT | F: AAGCCAAACGAAGGTCA |
| | R: AACCCATTTCAGCGATTGTC |
| zebrafish *mxc*-RT | F: GAGGCTTCACTTGGCAACTC |
| | R: TTGTTCCAATAAGGCCAAGC |
| zebrafish *phd3*-RT | F: CGCTGCGTCACCTGTATT |
| | R: TAGCATACGACGGCTGAACT |
| zebrafish *ldha*-RT | F: CCTTCTCAAGGATCTGACCG |
| | R: ACACTGTAATCTTTATCCGC |

| | |
|---|---|
| zebrafish *pdk1*-RT | F: TGAACCAGCACACTCTTCTG |
| | R: AGCATCTTTTACCACATCCG |
| zebrafish *vegfaa*-RT | F: TGCTCCTGCAAATTCACACAA |
| | R: ATCTTGGCTTTTCACATCTGCAA |
| zebrafish *glut1*-RT | F: GTGATTGGGTCCTTGCAGTT |
| | R: CTGAGAAGGAGCCGAGAATG |
| zebrafish *hif1αa*-RT | F: AGCCGCCACACTTTAGACAT |
| | R: CCTCTGGATCAAAACCCAAG |
| zebrafish *hif1αb*-RT | F: GCCACACTCTGGACATGAAG |
| | R: TCAAGAGGTCATCTGGCTCA |
| control siRNA | GGCUCCGUGUUAGACACCUUAGUGA |
| SVCV-G siRNA | GGCAGCCUGUGAUUCAGCCAUUUGA |
